# How sport participation affects older adults’ health—chain mediation based on intergenerational support and digital divide

**DOI:** 10.3389/fpubh.2025.1693987

**Published:** 2025-10-31

**Authors:** Jun-yi Zheng, Zong-wei Li, Meng-ding Liu, Mei Sun

**Affiliations:** ^1^School of Physical Education, Huazhong University of Science and Technology, Wuhan, China; ^2^School of Humanity and Law, Henan Agricultural University, Zhengzhou, Henan, China

**Keywords:** sport participation, older adults’ health, intergenerational support, digital divide, chain mediation effect

## Abstract

**Introduction:**

Sports participation is increasingly recognized as a key factor in promoting public health and addressing the challenges of population aging. However, the mechanisms linking sports participation to health outcomes, particularly through social and technological pathways, remain underexplored in China. This study aims to examine the direct and indirect effects of sports participation on older adults’ health, with a focus on the mediating roles of intergenerational support and the digital divide.

**Methods:**

Using data from the 2017 China General Social Survey (CGSS), a representative national dataset, we conducted regression analyses, path analysis, and bootstrap mediation tests. Variables included sports participation, intergenerational support, digital divide, and self-rated health, with controls for demographic and socioeconomic factors.

**Results:**

Sports participation significantly improved older adults’ health both directly and indirectly. Mediation analyses revealed three distinct pathways: (1) intergenerational support, (2) digital divide, and (3) a chain mediation effect whereby intergenerational support facilitated digital adaptation, which further enhanced health. The argument that sports participation is beneficial to health persists, although contextual factors such as urbanization and income levels may influence the extent of these benefits.

**Discussion:**

These findings highlight the importance of sports participation not only as a health-enhancing behavior but also as a catalyst for intergenerational interaction and digital inclusion. Policy efforts should strengthen family-based support systems and promote digital adaptation programs for older populations to maximize the health returns of sports participation.

## 1 Introduction

In 2021, the Central Committee of the Communist Party of China and the State Council issued the Opinions on Strengthening the Work on Aging, stating that sports participation constitutes a crucial pathway for actively addressing population aging and enhancing the social health support system for older people (1). Aligned with the strategic objectives outlined in the “Healthy China 2030” blueprint, several specific health targets are to be met by 2030, including 530 million regular participants in physical exercise, a 30% reduction in premature mortality from major chronic diseases among older people compared to 2015, and an average life expectancy of 79 years. Engagement in sports is considered a primary driver (2) for enhancing these indicators. The promotion of physical activity and health among older individuals has risen to the status of a national strategy, drawing heightened public attention. However, despite vigorous national efforts to develop senior sports, a pronounced knowing-doing gap persists at implementation, manifesting as a disconnect between policy intent and practical outcomes. The proportion of older people effectively and consistently engaging in physical activity remains significantly low, resulting in insufficient tangible health gains. This shortfall is particularly evident in the appropriateness of exercise environments, application of digital technologies, anticipation of risk factors, as well as social participation, all of which have failed to yield the anticipated effects. This implementation gap is further compounded by significant individual and environmental barriers faced by the older population. It includes a high prevalence of chronic conditions (e.g., sarcopenia, arthritis, and depression) (3), a lack of accessible and age-friendly sports facilities (4, 5), and psychological concerns such as the fear of falling (6). Consequently, this situation has emerged as a critical bottleneck impeding the national strategies for healthy aging. This study examines older people’s sports participation as a pathway to healthy aging. Employing a chain mediation model that integrates intergenerational support and the digital divide, it integrates socioecological theory, social support theory, and the technology acceptance model to analyze the mechanisms through which social governance fosters healthy aging.

Research on the correlation between sports participation and healthy aging initially focused on examining the impact on physiological indicators of bodily function in older people. Empirical studies have substantiated that sports participation exerts a significant influence (7) on improving health functions such as mitigating sarcopenia, delaying cognitive aging, and reducing the incidence of chronic diseases among the older people. Scholarly advancements have increasingly emphasized the multidimensional synergistic outcomes of sports participation, particularly evidenced in two critical domains: social connectedness and technological adaptability. For one thing, the social integration function within the older population has become increasingly prominent. There is a strengthening trend of social network embeddedness and intergenerational integration, accompanied by a significant enhancement in family collective efficacy. For another, research emphasizes leveraging technological feasibility to improve the social adaptability of older people. This progression enables a functional transition from rudimentary sports engagement to self-directed health governance, positioning health management optimization as a focus of research. Studies have found that intergenerational family physical activities (such as parent-child yoga, mutual-aid hiking, and commemorative group walks) strengthen affective intimacy bonds among family members. These activities not only effectively enhance the physical function of older people but also substantially alleviate depressive symptoms and feelings of loneliness in this demographic (8). Moreover, intergenerational family support can leverage a tiered technological empowerment framework to systematically mitigate the digital divide experienced by aging populations. It utilizes familial emotional bonds to improve exercise adherence among older people. The narrowing of the digital divide, in turn, enhances digital inclusion for the older people. This creates a compound enhancement effect for the health promotion outcomes of intergenerational support, ultimately forming a chain characterized by “social support and technological synergy.”

Based on the above theoretical propositions, this study constructs an explanatory model to elucidate the mechanisms through which sports participation influences older people’s health, using intergenerational support and the digital divide as mediating variables. This integrated framework advances beyond traditional single-mediation paradigms by addressing the knowledge-action gap in older people’s health management through family support mechanisms and technological empowerment mechanisms, offering theoretical and practical insights for healthy aging policies.

## 2 Theoretical basis

### 2.1 The impact of sports participation on older people’s health

Sports participation refers to goal-oriented and systematic physical practices implemented by individuals through physical exercise and recreational activities to enhance quality of life, safeguard physical and mental health, and promote social engagement (9). According to different forms and characteristics of participation, it is generally categorized into two types: direct participation involving personal engagement in physical exercise activities, and indirect non-practical participation involving leisure viewing of sports events (10). The theory of healthy aging argues that older populations often maintain high functional states and improve their health through regular physical exercise and social participation (11). Specifically, as an important leisure activity, sports participation has a protective effect on the overall health of the older people, enhancing their quality of life and facilitating comprehensive development through non-pharmaceutical interventions. Research indicates that sports participation can improve physiological functions in the older people, such as reducing the incidence of chronic diseases and delaying cognitive decline, while also meeting their social needs and fostering a healthy environment for the older people (3). However, the realization of health benefits from sports participation among the older people is constrained by the supportive roles of family support and technological environmental factors. According to the socio-ecological model, the promotion of overall health in older populations not only depends on individual motivation for behavioral change but also requires the synergy of resource accessibility among intergenerational family members and technological empowerment (12). This study thereby proposes Hypothesis 1: Sports participation can significantly and positively predict the health level of the older people.

### 2.2 Mechanisms of sports participation in older peoples’ health outcomes

#### 2.2.1 The mediating role of intergenerational support

As an informal form of social support, intergenerational support is a crucial manifestation of the social support system at the family level, serving as a key dynamic buffer in society. According to intergenerational support theory, adult children primarily provide family-based intergenerational support to the older people through financial assistance, daily care, and emotional support, helping them access better nutrition, overcome health challenges, and improve overall well-being (13). Research has found that, within the interactive pathways of family intergenerational support, collaborative support from intergenerational family members for older people’s sports participation yields significantly better health outcomes compared to the unidirectional “caregiver-care recipient” relationship (14).

In contemporary society, alongside the continuous evolution of traditional sports participation media technologies and the widespread use of smart wearable fitness devices, the core function of intergenerational support has shifted from resource transfer to responsive technological feedback. This shift involves co-building digital capital with the older people to facilitate their integration into a digital society. For instance, adult children may teach older adult family members how to operate smart fitness devices, remotely share health data, or calibrate online fitness guidance standards. These digital tools enable intergenerational health interventions, enhancing the self-efficacy and sustainability of older people’s sports participation, thereby positively impacting overall health. Longitudinal data reveal that older individuals receiving intergenerational support exhibit increased psychological capital accumulation, stronger health beliefs, and significantly higher overall health efficacy scores compared to control groups, demonstrating the transformative advantage of intergenerational health communities (15). Based on this, this study proposes Hypothesis 2: Sports participation may influence older people’s health through intergenerational support.

#### 2.2.2 The mediating role of the digital divide

The term “digital divide” originally referred to structural imbalances (16) in access to digital resources and communication technologies between economically disadvantaged and developed nations, rural and urban areas, and younger and older generations due to economic constraints, technological deficiencies, and educational divides. In this study, the digital divide specifically pertains to the older people digital divide. Compared to other groups, the older people face relative disadvantages in social status, economic conditions, and educational attainment, leading to significant disparities in digital tool awareness and practical digital skills. Specifically, the older people digital divide can be categorized into cognitive digital divide (subjective differences in older individuals’ understanding of digital tool functionalities), application digital divide (contextual behavioral variations among the older people in different digital technology usage scenarios), and skills digital divide (differences in the older people’s ability to master digital technology operations and technical competencies) (15). According to the Technology Acceptance Model, perceived usefulness and perceived ease of use are primary factors influencing individuals’ adoption and use of digital technologies. When individuals perceive digital technologies as effectively addressing their critical needs in specific task scenarios, the functional utility of these tools becomes optimal (17). Research indicates that the practical efficacy of sports participation can mitigate the technological exclusion mechanisms arising from the older people digital divide. For example, using fitness and health applications for consultation, progress tracking, data synchronization, and activity sharing can significantly reinforce healthy habits and promote overall well-being (18). Based on this, this study proposes Hypothesis 3: Sports participation may influence older people’s health by bridging the digital divide.

#### 2.2.3 The chain mediating effect of intergenerational support and digital divide

Intergenerational support and digital divide can form a continuous transmission effect, exerting hierarchical influence on older people’s health through a “social support and technological synergy” chain pathway. Older people’s sports participation (such as physical exercise and watching matches) stimulates cross-generational digital feedback behaviors from adult children. As key actors in family support, adult children assume crucial roles in facilitating older people’s digital socialization, including technology triggering, technological barrier elimination, and digital literacy construction. Research indicates that family support networks serve as effective focal points for bridging the older people digital divide. The closer the daily contact and interactive support among intergenerational members, the more intergenerational relationships can enhance older people’s acceptance of digital tool cognition and application, helping them overcome challenges like digital exclusion and digital divide (19).

The technology feedback behaviors from younger generations effectively reduce older people’s resistance and rejection toward smart fitness tools through technological cognition, capability cultivation, and behavioral reinforcement. By leveraging learning effects, these behaviors strengthen the technical application efficacy of digital sports management, ultimately achieving a closed-loop effect in promoting overall older people’s health (20). Studies demonstrate that enhancing older people’s digital socialization levels (including digital cognition, application, and skills) and narrowing or even bridging the older people digital divide significantly contribute to expanding social functions, deepening the integration of sports and older population care, and achieving healthy aging effects (3, 21). Based on this, this study proposes Hypothesis 4: In the process of sports participation affecting older people’s health, intergenerational support and digital divide may play a chain mediating role.

## 3 Research design

### 3.1 Data source

This study focuses on individuals aged 60 and above, utilizing data from the 2017 Chinese General Social Survey (CGSS 2017). The survey employs a stratified random sampling method, covering key dimensions such as individual health and behavior, family relationships, and digital literacy, which align closely with the research objectives. Complex survey or sample weights were not applied in the analysis. After data cleaning on critical variables, a final sample of 1,089 valid responses was obtained. Data analysis was conducted using SPSS 25.0.

### 3.2 Variable selection

The dependent variable is older people’s health. This study selects self-rated health to characterize the health status of older individuals, with specific measures being: “How would you rate your current physical health status?” and “In the past 4 weeks, how often has your health affected your work or daily activities?” Both questions use a 5-point scale with ascending scores, where the first question’s options are “Very unhealthy,” “Fairly unhealthy,” “Average,” “Fairly healthy,” and “Very healthy,” and the second question’s options are “Always,” “Frequently,” “Sometimes,” “Rarely,” and “Never.” Higher composite scores indicate better health status.

The core explanatory variable is sports participation. This study selects the frequency of exercise and watching sports live to characterize the intensity of older people’s sports participation. This item is reverse-scored to ensure data consistency—original scores of 1 and 5 are swapped, 2 and 4 are swapped, and 3 remains unchanged—to avoid statistical bias caused by question phrasing direction. The specific measures are: “In the past year, how often did you engage in physical exercise in your spare time?” and “In the past year, how often did you watch sports live in person in your spare time?” with options being “Never,” “A few times a year or less,” “A few times a month,” “A few times a week,” and “Daily.” Higher total scores indicate greater sports participation levels.

The mediating variables are intergenerational support and digital divide. Following Ding and Wang’s (22) research, reverse scoring is applied (processed the same way as the “sports participation” variable) to measure how frequently adult children provided assistance in the past year across three aspects: “financial support,” “help with household chores or caregiving,” and “listening to personal concerns,” with options including “Not at all,” “Rarely,” “Sometimes,” “Frequently,” and “Very frequently,” using the mean score of items as the variable score.

The digital divide variable references Li and Duan’s (15) research, characterizing it across three dimensions: cognitive, application, and skills. The cognitive digital divide investigates respondents’ attitudes toward the statement “A major advantage of the internet is enabling more people to access information,” specifically assessing their agreement levels regarding the internet’s roles in “political rights,” “public discourse,” “political understanding,” “helping officials better understand public opinion,” “accessing social resources,” “promoting social equity,” and “breaking down social stratification.” The application digital divide measures how frequently respondents used the internet in the past year for “social interaction,” “self-presentation,” “online activism,” “leisure and entertainment,” “information-seeking,” and “commercial transactions.” The skills digital divide uses reverse scoring (processed the same way as the “sports participation” variable) to measure respondents’ self-assessment of their competence in “using computers to browse websites,” “downloading apps on smartphones,” “searching for information online,” “verifying online information,” “expressing opinions online,” and “observing security during online payments.” All items are scored from 1 (“Strongly disagree”) to 5 (“Strongly agree”), with dimension scores calculated as the mean of respective items.

The control variables include gender, age (23), educational background, income, household registration (24), marital status, number of children, ethnicity, residence location, and social security enrollment. Table 1 reports the sample characteristics and assignment for all variables.

**Table 1:** Sample characteristics and assignment (*N* = 1,089).

Type	Variable name	*M ± SD*	Assignment/*n* (%)
Dependent variable	Health status	3.34 ± 0.87	1 = Very unhealthy … 5 = Very Healthy
Core explanatory variable	Sports participation	2.61 ± 0.84	1 = Never … 5 = Daily
Mediating variables	Intergenerational support	3.12 ± 0.81	1 = Never … 5 = Very Frequent
	Digital divide	3.09 ± 0.60	1 = Strongly disagree … 5 = Strongly Agree
Control variables	Gender	1.54 ± 0.50	1 = Male (496, 45.5%), 2 = Female (593, 54.5%)
	Age	1.63 ± 0.62	1 = Young-Old (60–74) (482, 44.3%), 2 = Old (75–89) (527, 48.4%), 3 = Oldest-Old (90+) (80, 7.3%)
	Education	1.98 ± 0.83	1 = No Formal education (294, 27.0%), 2 = Primary (602, 55.3%), 3 = Secondary (134, 12.3%), 4 = Higher Education (College) (43, 3.9%), 5 = Higher Education (Bachelor+) (16, 1.5%)
	Annual income	24525.09 ± 40597.21	Continuous variable
	Household registration	1.68 ± 0.83	1 = Agricultural (604, 55.5%), 2 = Non-Agricultural (231, 21.2%), 3 = Resident (251, 23.0%), 4 = Special populations (3, 0.3%)
	Marital status	2.26 ± 0.49	1 = Unmarried/cohabiting (26, 2.4%), 2 = Married (758, 69.6%), 3 = Not currently married (305, 28.0%)
	Number of children	2.34 ± 0.59	1 = None (23, 2.1%), 2 = 1 or 2 (651, 59.8%), 3 = 3 + (415, 38.1%)
	Ethnicity	1.06 ± 0.23	1 = Han (1,027, 94 0.3%), 2 = Minority (62, 5.7%)
	Residence area	1.33 ± 0.47	1 = Rural (727, 66.8%), 2 = Urban (362, 33.2%)
	Social security	1.93 ± 0.26	1 = Enrolled (81, 7.4%), 2 = Not enrolled (1,008, 92.6%)

All multi-item measures were drawn from CGSS 2017 and demonstrated acceptable internal consistency. Details of item wording, scoring, reverse coding, and reliability coefficients are provided in Appendix Table A1. Sports participation was measured by two items (exercise and spectating; *α* = 0.857, reverse coded), intergenerational support by three items (financial, household, emotional; *α* = 0.846, reverse coded), and the digital divide by three dimensions-cognition, application, and skills-with *α* values of 0.916, 0.881, and 0.889.

The digital divide was further modeled as a second-order construct. CFA results indicated standardized factor loadings of 0.702–0.794 for first-order items and 0.763–0.784 for the three dimensions on the higher-order factor. The second-order construct showed good reliability and validity (*α* = 0.932, CR = 0.809, AVE = 0.585), and overall model fit was satisfactory (*χ*^2^/df = 1.302, CFI = 0.994, IFI = 0.994, GFI = 0.974, RMSEA = 0.017), supporting the adequacy of the hierarchical structure.

## 4 Results and analysis

### 4.1 Correlation analysis

Pearson correlation analysis revealed that the four core variables—sports participation, intergenerational support, digital divide, and older people’s health—exhibited statistically significant positive correlations at the 1% level. In statistical terms, pairwise correlations among variables indicate that changes in one variable may, to some extent, predict trends in another, thereby providing a solid foundation for subsequent mediation effect testing in this study. Furthermore, the mean values of each variable in Table 2 show that sports participation was at a moderately low level, intergenerational support and older people’s health were at a moderately high level, while the digital divide fell within a moderate range.

**Table 2:** Correlation between main variables.

Variables	*M ± SD*	Sports participation	Intergenerational support	Digital divide	Older people’s health
Sports participation	2.61 ± 0.84	1			
Intergenerational support	3.12 ± 0.81	0.41^2)^	1		
Digital divide	3.09 ± 0.06	0.52^2)^	0.56^2)^	1	
Older people’s health	3.14 ± 0.87	0.41^2)^	0.41^2)^	0.56^2)^	1

Two decimals for coefficients, and ^1)^ *p* < 0.05, ^2)^ *p* < 0.01, the following table and figure is the same.

### 4.2 Baseline regression and path analysis

#### 4.2.1 Main effect test

Table 3 presents the baseline regression results incorporating control variables. Sports participation exhibited a statistically significant positive effect on older people’s health at the 1% level. Specifically, a one-unit increase in sports participation intensity was associated with a 0.42 improvement in older people’s health. These baseline regression results preliminarily confirm that higher sports participation intensity leads to better health outcomes among older people, thereby supporting Hypothesis 1.

**Table 3:** Regression results of sports participation on older people’s health.

Predictor variable	Older people’s health			95% CI	
	*β*	*SE*	*t*	Lower	Upper
Sports participation	0.42	0.03	14.53^2)^	0.36	0.48
Gender	0.03	0.05	0.62^1)^	0.07	0.13
Age	−0.05	0.04	−1.18^1)^	0.03	0.13
Education	0.03	0.03	0.86	−0.04	0.10
Annual income	0.00	0.00	−1.85	0.00	0.00
Household registration	−0.01	0.03	−0.32	−0.07	0.05
Marital status	0.03	0.05	0.60	−0.07	0.13
Number of children	−0.02	0.04	−0.37	−0.10	0.07
Ethnicity	0.03	0.11	0.29	−0.18	0.24
Residence area	0.06	0.06	0.98^1)^	0.06	0.17
Social security	−0.21	0.09	−2.28	−0.39	−0.03
*R^2^*	0.17				
*F*	20.62				

#### 4.2.2 Mediation effect test

Drawing on the bias-corrected percentile Bootstrap method proposed by Fang et al. (25), this study employed the PROCESS macro in SPSS 25.0 to construct 95% confidence intervals for mediation effects by resampling 5,000 iterations, while controlling for demographic variables such as gender, age, education background, and income.

**Figure 1:**
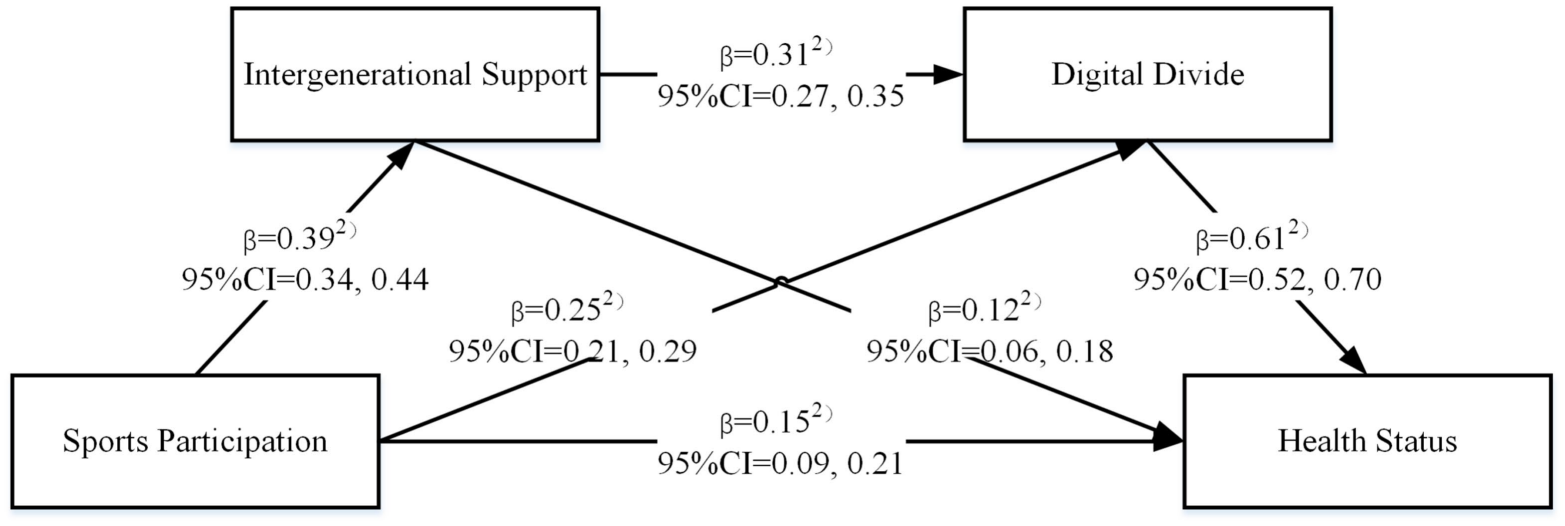
The chain mediation model.

The chain mediation regression analysis in Table 4 revealed that sports participation significantly and positively predicted older people’s health (*β* = 0.42, *p* < 0.001), confirming that higher sports participation intensity leads to better health outcomes among older people. Additionally, sports participation significantly and positively predicted intergenerational support (*β* = 0.39, *p* < 0.001). Both sports participation and intergenerational support, in turn, significantly and positively predicted the digital divide (*β* = 0.25, *p* < 0.001; *β* = 0.31, *p* < 0.001, respectively). Finally, after incorporating intergenerational support and the digital divide as mediators, sports participation remained a significant positive predictor of older people’s health (*β* = 0.15, *p* < 0.001), while both intergenerational support and the digital divide also significantly and positively predicted older people’s health (*β* = 0.12, *p* < 0.001; *β* = 0.61, *p* < 0.001, respectively). These findings indicate that intergenerational support and the digital divide serve as partial mediators in the relationship between sports participation and older people’s health. In other words, sports participation not only directly enhances older people’s health but also exerts an indirect influence through intergenerational support and the digital divide (see Figure 1 for the chain mediation model).

**Table 4:** Chain mediation regression analysis.

Regression equation		Overall fit indices			Regression coefficient significance			
Outcome variable	Predictor variable	*R*	*R^2^*	*F*	*β*	*SE*	*t*	95% CI
Older people’s health	Sports participation	0.42	0.17	20.62	0.42	0.03	14.53^2)^	(0.36, 0.48)
Intergenerational support	Sports participation	0.41	0.17	20.04	0.39	0.03	14.63^2)^	(0.34, 0.44)
Digital divide	Sports participation	0.65	0.42	66.25	0.25	0.02	13.86^2)^	(0.21, 0.29)
	Intergenerational support				0.31	0.02	16.51^2)^	(0.27, 0.35)
Older people’s health	Sports participation	0.36	0.34	42.98	0.15	0.03	4.76^2)^	(0.09, 0.21)
	Intergenerational support				0.12	0.03	3.66^2)^	(0.06, 0.18)
	Digital divide				0.61	0.05	12.79^2)^	(0.52, 0.70)

This study also examined the mediating mechanism through which intergenerational support and the digital divide operate in the relationship between sports participation and older people’s health (Table 5). The total effect of sports participation on older people’s health was 0.419 and statistically significant. Within this total effect, the direct effect was 0.146 (34.84% of the total effect), indicating that approximately one-third of sports participation’s impact on older people’s health occurs independently of mediators, while the indirect effect was 0.273, accounting for 65.16% of the total effect, mediated through intergenerational support and the digital divide.

**Table 5:** Chain mediation pathway analysis.

Pathway	Effect size	Proportion of total effect	Standard error	95% CI		Significance
				Lower	Upper	
Total effect	0.419	–	0.029	0.362	0.475	Significant
Direct effect	0.146	34.84%	0.031	0.086	0.206	Significant
Indirect effect	0.273	65.16%	0.020	0.235	0.314	Significant
Sports participation → Intergenerational support → Older people’s health	0.047	11.22%	0.013	0.021	0.074	Significant
Sports participation → Digital divide → Older people’s health	0.152	36.28%	0.015	0.124	0.183	Significant
Sports participation → Intergenerational support → Digital divide → Older people’s health	0.074	17.66%	0.009	0.058	0.092	Significant

Furthermore, regarding specific mediation pathways: The “sports participation → intergenerational support → older people’s health” mediation path effect value was 0.047 (accounting for 11.22% of total effect, confidence interval excluding zero); The “sports participation → digital divide → older people’s health” mediation path effect value was 0.152 (accounting for 36.28% of total effect, confidence interval excluding zero); The “sports participation → intergenerational support → digital divide → older people’s health” chained mediation path effect value was 0.074 (accounting for 17.66% of total effect, confidence interval excluding zero). These results demonstrate that all pathways exert statistically significant effects in the mechanism linking sports participation to older people’s health outcomes. Collectively, the findings validate Hypotheses 2, 3, and 4.

#### 4.2.3 Robustness check

To further verify the reliability of the empirical results, an additional robustness analysis was performed by adjusting the sequential order of mediating variables in the chain mediation model. Specifically, two alternative model structures were tested: in the first, sport participation influences older adults’ health through the digital divide followed by intergenerational support; in the second, the sequence of the two mediators was reversed. The comparative estimation results revealed that the coefficients, significance levels, and indirect effects remained largely unchanged across both specifications (Table 6). This finding indicates that variations in the assumed causal sequence did not substantially alter the direction or magnitude of the mediating effects. Overall, the results demonstrate that the empirical conclusions of this study are robust to alternative model settings and provide further support for the internal consistency and validity of the analysis.

**Table 6:** Robustness test regression results.

Regression equation		Overall fit indices			Regression coefficient significance			
Outcome variable	Predictor variable	*R*	*R^2^*	*F*	*β*	*SE*	*t*	95% CI
Older people’s health	Sports participation	0.42	0.17	20.62	0.42	0.03	14.53^2)^	(0.36, 0.48)
Digital divide	Sports participation	0.53	0.28	37.94	0.37	0.02	20.14^2)^	(0.34, 0.41)
Intergenerational support	Sports participation	0.58	0.34	45.71	0.15	0.03	5.32^2)^	(0.09, 0.20)
	Digital divide				0.65	0.04	16.51^2)^	(0.57, 0.73)
Older people’s health	Sports participation	0.36	0.34	42.98	0.15	0.03	4.76^2)^	(0.09, 0.21)
	Digital divide				0.61	0.05	12.79^2)^	(0.52, 0.70)
	Intergenerational support				0.12	0.03	3.66^2)^	(0.06, 0.18)

## 5 Discussion

### 5.1 The impact of physical activity participation on older people’s health

Within older people’s health management factors, physical activity participation is widely recognized as yielding significant health benefits. Evidence suggests that increasing numbers of older people are becoming aware of both the personal advantages and positive systemic health effects associated with regular physical activity (26). According to healthy aging theory, this study postulated that physical activity participation would serve as a significant positive predictor of older people’s health outcomes—a hypothesis substantiated by our data analysis and consistent with established research findings (27).

Engagement in physical activity constitutes a vital mechanism for attaining healthy aging outcomes. Older people who maintain long-term exercise regimens characterized by both high frequency and extended duration consistently demonstrate superior self-rated health assessments (28). Concurrently, psychological factors, particularly the fear of falling and associated injuries, can lead to activity avoidance. These challenges are often compounded by environmental barriers such as a lack of safe, accessible, and well-maintained public spaces and sports facilities, which limit the potential health gains and exacerbate the knowing-doing gap. Primary motivations for exercise participation among the older population include enhancing “physical constitution,” “disease prevention,” and “maintaining vitality” (6)—motivations that further underscore the fundamental role of physical activity in promoting health among older people. Comparably, analysis reveals that older people exhibit a marked preference for physical activity over alternative social engagements as a means to preserve and expand social networks, fulfill psychological needs, and facilitate comprehensive physical-mental wellbeing (4, 5).

### 5.2 The mediating role of intergenerational support

The findings demonstrate that intergenerational support plays a partial mediating role in the relationship between physical activity participation and older people’s health outcomes. Older individuals’ engagement in physical activities not only directly enhances health status but also indirectly improves health by increasing intergenerational support. This mechanism aligns with Burnett et al.’s (29) proposition that physical activity facilitates intergenerational bonding, helping older people strengthen family and social interactions, reduce feelings of loneliness and depression, fulfill self-actualization needs, and ultimately promote health while preventing disability (30). Particularly in informal interactions, the intergenerational support provided by adult children serves as a primary channel for older individuals to access social resources (31), exerting significant influence on their health.

While most older individuals increasingly recognize the health benefits of physical activity participation, additional sports-related expenditures and family responsibilities often create barriers to sustained participation (32). For one thing, limited incomes lead older people to prefer low-cost activities like walking and square dancing (33). However, as health awareness and quality of life expectations rise, the diversified fitness needs of the older people inevitably create financial pressures. In this context, children’s financial support—such as purchasing sports equipment or covering fitness class fees may help alleviate the economic burden of physical activity participation. For another, traditional values often lead older individuals to prioritize caregiving responsibilities over personal exercise (34, 35), while emotional exchanges and cooperation with children can significantly enhance seniors’ sense of identity and resource acquisition capacity (36), thereby supporting sustainable physical activity participation.

Collectively, high levels of physical activity participation create more opportunities for intergenerational interaction, motivating children to actively facilitate suitable exercise conditions for their older adult parents. This may include providing financial support to improve material conditions, monitoring post-exercise physical conditions with appropriate care, and enhancing psychological comfort through exercise-related conversations. Such support boosts older individuals’ motivation for physical activity and overall wellbeing, leading to better health outcomes (37). Essentially, Elevated sports participation effectively supplements older people’s health needs (38) by enhancing intergenerational support systems while simultaneously boosting self-efficacy (39).

### 5.3 The mediating role of the digital divide

In examining the relationship between sports participation, the digital divide, and the health of older people, it has been demonstrated that sports participation positively enhances older people’s health by bridging the digital divide, which aligns with the conclusions of this study that older people who engage in sports activities establish mechanisms for social support, technological practice, and data feedback. These mechanisms systematically mitigate cognitive, applicational, and skill-based dimensions of the digital divide, thereby amplifying the empowering effect of digital inclusion on overall health promotion.

According to the “network enhancement” theory, sports participation possesses distinct social attributes. In terms of technological cognitive restructuring, older people who engage in physical activities demonstrate significantly higher acceptance and awareness of health-related apps compared to non-participants, and they exhibit a greater tendency to interact with digital technologies through sports participation (40). This process aligns with the Technology Acceptance Model (TAM), where the social nature of sports activities substantially increases the frequency of digital tool usage among older people while reducing cognitive resistance toward such technologies (41). This phenomenon underscores the effectiveness of exercise-embedded scenarios in facilitating technological cognitive restructuring.

Regarding the reinforcement of practical application, a significant positive correlation exists between the frequency of sports participation and older people’ digital application proficiency, particularly in health management-related applications (42). Physical activities provide older people with high-frequency opportunities for technological application, facilitating a shift in digital skills from passive reception to active utilization. For instance: Smart fitness trails and health-monitoring wearables collect real-time exercise data (e.g., heart rate, step count), integrating AI algorithms to generate personalized fitness plans; structured digital exercise programs (e.g., guided Tai Chi video sessions) employ phased goal-setting (e.g., unlocking new movement modules weekly). Such digital health management models not only enhance exercise outcomes but also strengthen older people’ sense of control over their own health, consistent with the self-efficacy enhancement mechanism in cognitive behavioral theory (43).

In terms of technological skill improvement and health promotion, older people participating in digitally empowered sports activities exhibit positive trends in physical and mental well-being, reduced healthcare burdens, and enhanced social interactions (44).

In summary, physical activity serves as a practical context for enhancing digital literacy by mitigating age-related exclusion in technology adoption, thereby transforming digital health resources into tangible health capital. Through sports participation, older people gain access to online platforms where they can acquire scientifically validated exercise knowledge, select age-appropriate physical activities, and integrate into virtual fitness communities—all of which provide enhanced motivation and social support for sustained engagement.

This sports-mediated model of digital empowerment not only helps older people bridge the digital divide and achieve health autonomy through evidence-based exercise, but also reinforces their health awareness and self-management capacity. Consequently, it creates a more efficient method for attaining health objectives (45).

### 5.4 The chain mediation effects of intergenerational support and digital divide

The study further reveals that intergenerational support and the digital divide collectively serve as chain mediators in the relationship between sports participation and older people’s health outcomes. Existing research on intergenerational support’s impact on older people’s health primarily focuses on dimensions such as emotional support (46), financial support (47), psychological support (48), and domestic assistance (13). Meanwhile, studies examining the digital divide’s influence on older people’s health have concentrated on psychological efficacy, social support, technological intervention, and digital ethics (49).

This study systematically integrates multidimensional analytical frameworks of intergenerational support and the digital divide, providing deeper insights into the intrinsic mechanisms through which sports participation affects older people’ overall health. Cross-sectional statistical data empirically validate specific pathways of this chain mediation effect. Specifically, sports participation enhances social capital among older populations through intergenerational support. Family-based intergenerational interactions—including emotional feedback, resource transfer, informational support, and reverse technology mentoring—effectively address older people’ dependency needs while expanding their relational network capital. This process subsequently alleviates digital adaptation barriers caused by cognitive anxiety or technological resistance, ultimately enhancing health promotion efficacy in older populations. These findings confirm the synergistic interaction between intergenerational support and the digital divide, highlighting the digital adaptation effect facilitated by intergenerational support. The results provide a novel theoretical perspective for comprehensively analyzing the complex determinants influencing health outcomes in aging populations.

Furthermore, the specific mechanism of the chain mediation effect involving intergenerational support and the digital divide manifests primarily in the following ways: physical activity participation, particularly family-based joint exercise, effectively activates intergenerational interaction resources. The emotional support dimension enhances older people’ subjective willingness to engage in physical activities, while the economic support dimension—such as providing technological or informational resources—improves the scientific rigor and standardization of their participation. The daily care support dimension reinforces the sustainability of their physical activity engagement, collectively forming a health-enhancing chain reaction characterized by “behavioral triggering, resource activation and health transformation.” “Within this framework, the tripartite structure of intergenerational support serves as a buffering layer against the digital divide in how physical activity influences older people’s health outcomes. Older people with higher levels of physical activity participation are better able to strengthen their digital embeddedness through family intergenerational support—such as using smart fitness devices, accessing online exercise guidance, and participating in virtual fitness classes—thereby fostering a virtuous cycle of improved health outcomes. Moreover, as older people progressively narrow or even bridge the digital divide, the sustainability of their physical activity participation, exercise adherence, and health benefits significantly increase. This not only consolidates the quality of intergenerational support and reinforces intergenerational health solidarity but also leads to a marked improvement in their digital health literacy and overall health status. This chain mediation mechanism fully embodies the intergenerational digital synergy model for health promotion, empirically validating the strategic coupling effect of intergenerational support and digital empowerment in enhancing the health of older people. It provides a reference for intervention strategies aimed at improving health outcomes in China’s aging society.

## 6 Conclusion

This study empirically examines the influence of control variables—including gender, age, education level, annual income, household registration, marital status, number of children, ethnicity, place of residence, and social security participation—on the overall health status of older people. The findings reveal significant health disparities among older people, with widening gaps under different demographic and socioeconomic conditions. Specifically, male older people exhibit better overall health than females, while health status declines markedly with advancing age, particularly among the oldest-old. Socioeconomic factors such as education, income, urban or rural residence, and social security enrollment disproportionately affect middle-aged and younger-old people, with urban residents demonstrating higher physical activity participation and self-rated health than their rural counterparts. These results highlight the necessity of addressing health heterogeneity among older people through tailored interventions that prioritize equity in healthcare access and resource allocation. A stratified, precision-based policy approach is essential to mitigate disparities, enhance service accessibility, and foster a supportive environment for healthy aging, ultimately contributing to social stability and sustainable development. Future research should further explore longitudinal health trajectories and the intersectional effects of socioeconomic determinants to refine intervention strategies.

Secondly, this study validates the pathways and mechanisms through which physical activity participation, intergenerational support, the digital divide, and older people’s health outcomes interact. Empirical findings demonstrate that physical activity participation positively influences both intergenerational support and older people’s health outcomes, indicating that direct or indirect engagement in sports activities enhances social participation among older people, thereby strengthening family intergenerational interactions and balanced support systems. This process creates a more robust intergenerational health reserve, significantly improving overall health and well-being among the older people. Besides, intergenerational support positively affects older people’s health outcomes by mitigating the digital divide, as technological reverse mentoring embedded in intergenerational resource support enhances older people’ digital literacy and adaptive capacity. This assistance helps them overcome challenges posed by digital transformation while simultaneously improving their quality of life through emotional and caregiving support, ultimately elevating their health status.

Thirdly, this study reveals the significant mediating roles of intergenerational support and the digital divide in linking physical activity to older people’s health outcomes. Cross-generational companionate sports activities strengthen intergenerational support mechanisms, which then promote health efficacy through exercise supervision, digital resource provision, and health knowledge transmission. Concurrently, the digital divide mediates this relationship as older people gradually develop digital competencies, such as using smart fitness devices and participating in online health assessments to overcome technological barriers and achieve precise health improvements. Crucially, intergenerational support and the digital divide jointly operate as a sequential mediation chain: physical activity enhances intergenerational support, which subsequently reduces digital disparities, ultimately elevating overall health levels. This constructs a “behavioral activation, relational reinforcement and technological empowerment” pathway. The chain mediation mechanism underscores the necessity of transcending conventional unidimensional interventions by developing multidimensional models that integrate intergenerational solidarity and digital inclusion, thereby optimizing healthy aging efficacy.

Fourthly, this study systematically elucidates the synergistic mechanism through which intergenerational support and the digital divide jointly influence the relationship between physical activity participation and older people’s health outcomes. This provides practical insights for facilitating social integration among older people and addressing the dual challenges of technological barriers and support deficiencies in their social participation. By establishing a comprehensive three-dimensional analytical framework encompassing family, society, and technology, this research demonstrates how intergenerational digital empowerment enhances the health-promoting effects of physical activity by bridging technological gaps. These findings offer significant value for advancing active aging strategies and developing health-friendly social environments for the older population.

Nevertheless, this study has several limitations that should be considered. First, the actual pathways through which physical activity influences older people’s health are inherently complex. While this study identified core variables and key mediation pathways based on social support theory and technology acceptance models, it did not fully account for potential moderating variables such as social support networks, social identity, demographic capital, and intrinsic motivation. Future research should refine the analytical model to improve its explanatory power by incorporating these factors. Second, the regional comparative analysis of sample data could be strengthened. Although the data were drawn from the China General Social Survey (CGSS 2017) and covered major provinces across China, subsequent studies could enhance the robustness of findings by incorporating comparative analyses between eastern, central, and western regions—particularly focusing on areas with inadequate digital infrastructure and structural resource deficiencies to better understand intergenerational compensatory effects. Additionally, the assessment of overall health status in this study relied on subjective self-reports. Future research could improve measurement accuracy by incorporating objective monitoring data from smart fitness devices and specific health indicators to strengthen the scientific rigor of core variable assessment.

## Data Availability

The datasets presented in this study can be found in online repositories. The names of the repository/repositories and accession number(s) can be found at: http://cgss.ruc.edu.cn/English/Home.htm.
