# Supplementary material for: How sport participation affects older adults’ health—chain mediation based on intergenerational support and digital divide
Source: Front Public Health. 2025 Oct 31;13:1693987. doi: 10.3389/fpubh.2025.1693987 (PMC12616862; doi:10.3389/fpubh.2025.1693987)
Supplement: Supplementary file 1 [file Table_1.docx]

**Appendix Table 1. Measurement Items, Scoring Rules, and Reliability/Validity Results for Key Constructs**

| **Variable** | **Items / Standardized Factor Loadings** | **Scoring Rules** | **Reverse Coding** | **Cronbach’s α** | **CR** | **AVE** |
| --- | --- | --- | --- | --- | --- | --- |
| **Older Adults’ Health** | How would you rate your current physical health status? (0.899)  In the past four weeks, how often has your health affected your work or daily activities? (0.881) | Five-point scale: 1 = Very unhealthy / Always … 5 = Very healthy / Never; mean score | None | 0.884 | 0.884 | 0.792 |
| **Sport Participation** | In the past year, how often did you engage in physical exercise in your spare time? (0.836)  In the past year, how often did you watch sports live in person in your spare time? (0.898) | Five-point scale: 1 = Never … 5 = Daily; mean score | All items reverse-coded (1↔5, 2↔4, 3 unchanged) | 0.857 | 0.859 | 0.753 |
| **Intergenerational Support** | Did your children provide financial support? (0.790)  Did your children help with household chores or caregiving? (0.814)  Did your children listen to your personal concerns or thoughts? (0.810) | Five-point scale: 1 = Not at all … 5 = Very frequently; mean score | All items reverse-coded (1↔5, 2↔4, 3 unchanged) | 0.846 | 0.846 | 0.648 |
| **Digital Divide** | **Cognitive Dimension** (Std. FL: 0.763)  The internet enables more people to access information. (0.741)  The internet grants people more political rights. (0.759)  The internet allows more public discussion of government affairs. (0.741)  The internet helps the public better understand politics. (0.771)  The internet helps officials better understand public opinion. (0.783)  The internet provides more access to social resources. (0.775)  The internet promotes social equity. (0.770)  The internet breaks down social stratification. (0.740) | Five-point scale: 1 = Strongly disagree … 5 = Strongly agree; mean score | None | 0.916 | 0.916 | 0.578 |
|  | **Application Dimension** (Std. FL: 0.784)  In the past year, how often did you use the internet for:  Social interaction (e.g., chat tools). (0.732)  Self-presentation (e.g., social media, blogs). (0.743)  Online activism (e.g., rights protection, advocacy). (0.702) Leisure/entertainment (e.g., games, music, videos). (0.765)  Information seeking (e.g., searching, browsing news). (0.758)  Business transactions (e.g., transfers, payments, shopping). (0.770) | Five-point scale: 1 = Never … 5 = Always; mean score | None | 0.881 | 0.882 | 0.556 |
|  | **Skills Dimension** (Std. FL: 0.748)  Please indicate whether the following apply to you:  I can use a computer to browse websites. (0.739)  I can download and install apps on a smartphone. (0.749)  I can easily find information online. (0.741)  I verify forwarded messages online before believing them. (0.757)  I know how to express my opinions online. (0.755)  I pay attention to security when making online payments. (0.794) | Five-point scale: 1 = Strongly disagree … 5 = Strongly agree; mean score | All items reverse-coded (1↔5, 2↔4, 3 unchanged) | 0.889 | 0.889 | 0.572 |
| **Digital Divide**  **(Second-order construct)** | Three first-order dimensions loadings:  cognition (0.763), application (0.784), skills (0.748) | | | 0.932 | 0.809 | 0.585 |
| **Overall Model Fit** | χ²/df = 1.302, CFI = 0.994, IFI = 0.994, GFI = 0.974, RMSEA = 0.017 | | | | | |

Note: All items were derived from the CGSS 2017 survey, with values in parentheses indicating standardized factor loadings. Older adults’ health, sport participation, and intergenerational support were treated as composite indicators, while the digital divide was modeled as a latent construct with both first- and second-order CFA results; reverse-coded items were recoded prior to analysis.
